# Pressure-induced structural and spin transitions of Fe_3_S_4_

**DOI:** 10.1038/srep46334

**Published:** 2017-04-12

**Authors:** Shengxuan Huang, Duan Kang, Xiang Wu, Jingjing Niu, Shan Qin

**Affiliations:** 1 Key Laboratory of Orogenic Belts and Crustal Evolution, MOE, Peking University and School of Earth and Space Sciences, Peking University, Beijing 100871, P. R. China; 2 State key laboratory of geological processes and mineral resources, China University of Geosciences (Wuhan), 430074, P. R. China

## Abstract

Greigite (Fe_3_S_4_), isostructural with Fe_3_O_4_ has recently attracted great scientific interests from material science to geology due to its complicated structure and electronic and magnetic configurations. Here, an investigation into the structural, magnetic and electronic properties of Fe_3_S_4_ under high pressure has been conducted by first-principle calculations based on density functional theory. The results show that a first-order phase transition of Fe_3_S_4_ would occur from the inverse spinel (SP) structure to the Cr_3_S_4_-type (CS) structure at 3.4 GPa, accompanied by a collapse of 9.7% in the volume, a redistribution of iron cations, and a half-metal to metal transition. In the CS-Fe_3_S_4_, Fe^2+^ located at octahedral environment firstly undergoes a transition from high-spin (HS) state to low-spin (LS) state at 8.5 GPa and Fe^3+^ subsequently does at 17 GPa. The Equation of State for different phases of Fe_3_S_4_ are also determined. Our results not only give some clues to explore novel materials by utilizing Fe_3_S_4_ but also shed light on the fundamental information of Fe_3_O_4_, as well as those of other SP-AB_2_X_4_ compounds.

Spinel-structured (SP) AB_2_X_4_ (X = O, S, Se) compounds with space group 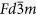 (Z = 8) [see Supplementary Fig. S1(a)], such as MgAl_2_O_4_, FeCr_2_O_4_, Fe_3_O_4_ and FeCr_2_S_4_, have attracted great scientific interests from geophysics to material science. For example, the phase transition of (Mg,Fe)_2_SiO_4_ from olivine to wadsleyite, and subsequently to ringwoodite explains the seismic discontinuity in 410 km and 520 km in Earth’s interior, respectively^1^,^2^. In addition, the discovery of a new class of multiferroic materials, in which ferroelectricity and ferromagnetism, as well as other unusual physical properties are compatible with a cubic spinel symmetry holds promise for new generations of functional materials^3^,^4^. These significant physical phenomena are possibly attributed to intimate coupling between structural, electronic and magnetic properties in the material^5^,^6^.

High pressure, as one important thermodynamic parameter, can strongly affect a material’s structures and electronic and magnetic configurations, which inspires a renewed interest in the fundamentally physical and chemical properties of spinel-structured AB_2_X_4_ compounds. Besides dissociating into the assemblage of simpler compounds, three orthorhombic phases of CaTi_2_O_4_-type (space group: *Bbmm*, Z = 4, CT), CaMn_2_O_4_-type (space group: *Pbcm*, Z = 4, CM) and CaFe_2_O_4_-type (space group: *Pnma*, Z = 4, CF) structures have been identified by both experiments and theoretical calculations as high-pressure polymorphs of oxide spinels with the initial chemical formula^7^,^8^,^9^,^10^,^11^,^12^. In addition, some of them can experience the *I*4_1_/*amd* structure, a subgroup of the SP structure, before transforming into the denser orthorhombic structures^13^,^14^. While for thiospinels and selenospinels, the phase transition to a Cr_3_S_4_-type (space group: *I*2/*m*, Z = 2, CS) structure upon compression has been confirmed as well^15^,^16^.

In particular, the high-pressure behavior of magnetite (Fe_3_O_4_), an inverse spinel, is fundamentally important for understanding the oxidation state of Earth’s interior. A phase transition of Fe_3_O_4_ to a monoclinic structure was observed at ~25 GPa, but more details about the high-pressure structure, such as space group and atomic positions were not provided^17^. Later, both CM-type and CT-type structures were proposed to be candidates for the high-pressure post-spinel phase of Fe_3_O_4_^8^,^9^,^18^,^19^. However, the accurate high-pressure polymorph of Fe_3_O_4_ remains unclear. Additionally, the electronic and magnetic behaviors of Fe_3_O_4_ under extreme conditions are under debate. Transformation of the electron charge density from the octahedral to the tetrahedral site of Fe_3_O_4_ results in a phase transition to a normal spinel structure that was proposed at ~7 GPa^20^, but data obtained by high-pressure single crystal X-ray diffraction experiments did not support such a conclusion^21^. The amplitude of the Fe *K*-edge X-ray magnetic circular dichroism (XMCD) signal of Fe_3_O_4_ was observed to decrease discontinuously by 50% between ~12 and ~16 GPa, which was interpreted by a high-spin (HS) to intermediate-spin (IS) transition of Fe^2+^ on the octahedral sites^22^. However, Baudelet *et al*.^23^ found neither an inverse-to-normal phase transition nor a spin transition in Fe_3_O_4_ up to ~41 GPa by XMCD experiments. On the other hand, using first-principle calculation, Ju *et al*.^24^ predicted a very complicated spin transition sequence from HS state to IS state and further to low-spin (LS) state of iron above 30 GPa. Another calculation found that the HS state of iron was stable at least up to 45 GPa instead^25^. Therefore, there exist significant controversies on high-pressure polymorphs and electronic and magnetic properties of compressed Fe_3_O_4_, which restrain our understanding of oxide buffer and magnetic field involving Fe_3_O_4_ in the lower mantle.

Since various structural, electronic and magnetic transitions of Fe_3_O_4_ can only be observed at very high pressure that are challenging for accurate detections, the high-pressure studies of compounds analogous to Fe_3_O_4_, that may experience similar phase transitions at relatively low pressure conditions, are urgent. Greigite (Fe_3_S_4_) is isostructural with Fe_3_O_4_ and can be considered as the sulfide counterpart of Fe_3_O_4_. Hence, Fe_3_S_4_ is a good candidate for investigations of the high-pressure behavior of Fe_3_O_4_. In addition, Fe_3_S_4_ has been reported to play an important role not only on hydrogen storage^26^,^27^, lithium-ion batteries^28^ and water dissociation^29^,^30^ but also on paleomagnetism^31^,^32^. The saturation magnetization of Fe_3_S_4_ with ferrimagnetic (FIM) property was reported in a wide range of values from 1.06 to 3.35 *μ*_*B*_/f.u., which was attributed to different samples used in the experiments^33^,^34^,^35^,^36^,^37^. However, any result on the magnetic moment is much lower than that of Fe_3_O_4_ (~4.0 *μ*_*B*_/f.u.), attributed to an increased degree of covalence between iron and sulfur compared to oxygen ligands or by greater delocalization of the 3*d* electrons in Fe_3_S_4_^38^. An interesting phenomenon, the Verwey transition was observed in Fe_3_O_4_, where the resistivity abruptly increased implying a metal-to-insulator transition by charge ordering when the temperature decreased to ~120 K^39^. However, neither low-temperature magnetic nor structural transition was evident for Fe_3_S_4_ by high-resolution neutron powder diffraction (NPD) and polarized neutron diffraction (PND)^38^. Later, two research groups reported opposite results about whether or not the Verwey transition exists in Fe_3_S_4_ by first-principle calculations^40^,^41^. Generally speaking, the structural, electronic and magnetic properties of Fe_3_S_4_ are also controversial and more complex than we have expected. However, to the best of our knowledge, no work has been published about the high-pressure behavior of Fe_3_S_4_. Hence, our study aims to explore the structural stability and electronic and magnetic configurations of Fe_3_S_4_ under high pressure by first-principle calculations based on density functional theory (DFT), which could provide more fundamental details about the physical and chemical properties of Fe_3_S_4_ and aid to our understanding of the high-pressure behavior of Fe_3_O_4_.

## Results

**Figure 1:**
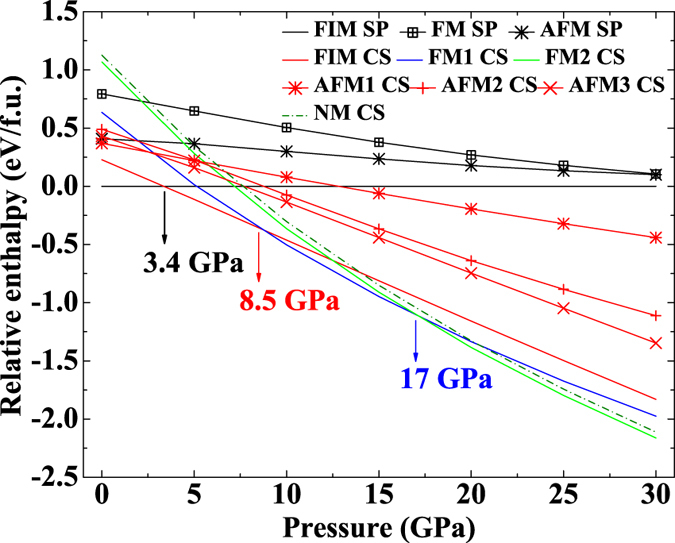
Static enthalpy differences among various candidate phases as a function of pressure. FIM, FM, AFM and NM represent ferrimagnetic, ferromagnetic, antiferromagnetic and nonmagnetic structures, respectively.

The corresponding Equation of State (EoS) parameters of different Fe_3_S_4_ phases are listed in Table 1. Based on those parameters, we calculate static enthalpy differences among various candidate phases as a function of pressure (Fig. 1). The FIM-SP phase with the lowest enthalpy is the ground state at ambient pressure in agreement with previous studies^38^,^40^. Upon compression, the CT-, CM-, CF-type Fe_3_S_4_ are unstable under all calculated pressure range no matter what types of magnetic structures they are, which indicates that the phase transition from the spinel structure to orthorhombic structures is energetically unfavorable up to at least 30 GPa. A structural phase transition from the SP structure to the CS structure keeping ferrimagnetic is predicted to occur at 3.4 GPa and 0 K. The transition point is relatively low compared with ~20 GPa in Fe_3_O_4_, and more similar to ~7 GPa in FeCr_2_S_4_^15^. Another two phase transitions are predicted to occur at 8.5 GPa and 17 GPa, respectively due to spin state transitions, which will be discussed later.

**Figure 2:**
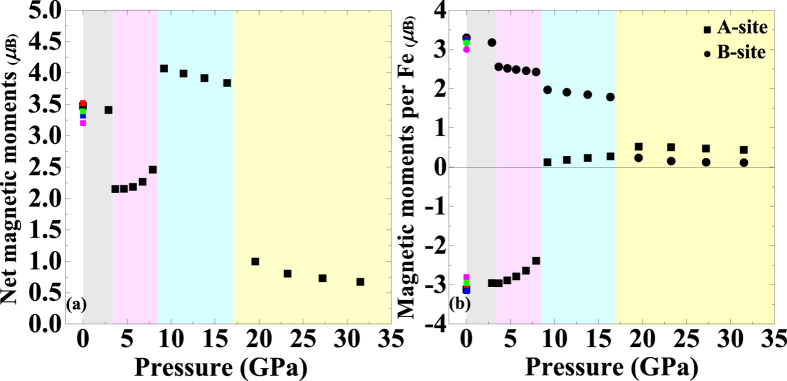
Calculated net magnetic moments (**a**) and sub-lattice magnetic moments per Fe of A-site and B-site (**b**) as a function of pressure. The magnetic moments of Fe on the B-site are defined as positive and therefore those of Fe on the A-site in the FIM-SP and FIM-CS phases are negative. The grey, pink, blue and yellow regions represent FIM-SP, FIM-CS, FM1-CS and FM2-CS phases, respectively. The previous data marked with red (experimental results, Exp.), blue (Exp.), green (theoretical calculations, The.) and pink (The.) points are extracted from refs 38,40 and 42, respectively for comparison.

Figure 2 displays the net and individual sub-lattice magnetic moments as a function of pressure, respectively, where the available experimental and theoretical data at ambient pressure are included for comparison^38^,^40^,^42^. The calculated sub-lattice magnetic moments of SP-type Fe_3_S_4_ at ambient pressure are −3.12 *μ*_B_ (m_A_) and 3.30 *μ*_B_ (m_B_), indicating a ferrimagentic structure in agreement with the data obtained by NPD^38^. This result suggests that the simulation is well improved by the DFT + *U* method (see Supplementary text and Figure S2). The calculation also presents HS electronic configurations for Fe sites, where the effective spin in the tetrahedral site is lower than that in the octahedral one. The sub-lattice magnetic moments on both sites decrease with the increasing pressure. However, the net magnetic moment of FIM-CS Fe_3_S_4_ increases upon compression from 3.4 GPa to 8.5 GPa because the magnetic moment of Fe_A_ decreases more rapidly than Fe_B_ does. At 8.5 GPa, the ferromagnetic (FM) CS phase with LS Fe_A_ and HS Fe_B_ (named as FM1-CS) becomes energetically favorable compared with the FIM-CS phase, accounting for a significant increase in the net magnetic moment which is even higher than that at ambient pressure. Subsequently, a pressure-induced HS-LS transition of Fe_B_ in the FM-CS phase (named as FM2-CS) results in a large drop in the net magnetic moment above 17 GPa. Under further compression, the net magnetic moment and sub-lattice magnetic moments decrease slowly without any abrupt changes up to 30 GPa.

**Figure 3:**
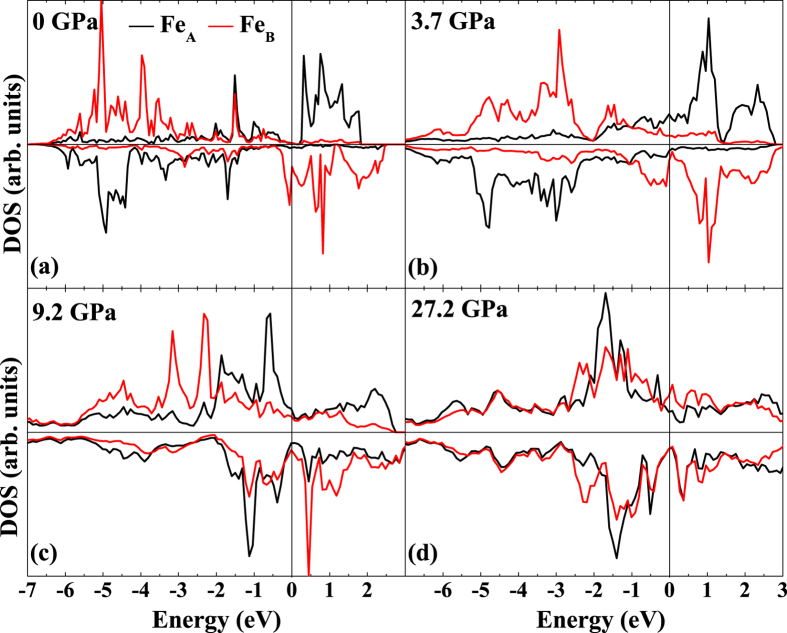
Calculated Fe DOS of each equivalent atom site of different Fe_3_S_4_ phases by GGA + *U* methods. (**a**) DOS of Fe in FIM-SP phase at 0 GPa, (**b**) DOS of Fe in FIM-CS phase at 3.7 GPa, (**c**) DOS of Fe in FM1-CS phase at 9.2 GPa and (**d**) DOS of Fe in FM2-CS phase at 27.2 GPa. The Fermi level is indicated by the vertical line in each DOS.

The electronic density of states (DOS) of Fe at each equivalent site in four phases (FIM-SP, FIM-CS, FM1-CS and FM2-CS) are shown in Fig. 3. In the FIM-SP phase [Fig. 3(a)], both Fe_A_ and Fe_B_ are identified in HS state. The DOS in the spin-down state of Fe_B_ crosses the Fermi level while there is a gap of 0.2 eV in the spin-up state of Fe_A_, which is in agreement with similar theoretical studies^40^. Besides, it is commonly accepted that the DFT calculation systematically underestimates the band gaps of materials. Therefore, the half-metallic character for FIM-SP Fe_3_S_4_ at ambient pressure could be confirmed by our calculation. At 3.7 GPa, the DOS of the spin-up state of Fe_A_ on the bottom of conduction bands changes significantly, indicating a variation of local environment of Fe_A_ due to the SP-CS phase transition. It is obvious that the contribution of electrons of Fe to the top of valance bands in the high-pressure phase [Fig. 3(b)] is more pronounced than that in the low-pressure phase [Fig. 3(a)], contributing to the metallic property of the FIM-CS phase. However, whether or not FIM-CS Fe_3_S_4_ becomes metallic upon compression needs further experimental verification due to the underestimation of band gaps of materials by DFT calculations as mentioned above. At 9.2 GPa [Fig. 3(c)], the orbitals in the spin-up state of Fe_A_ in the FM1-CS phase mainly form the top of valance bands, which could be interpreted as the HS-LS transition of Fe_A_. While the DOS in the spin-down state of Fe_B_ does not change significantly, suggesting that Fe_B_ remains the HS state under such conditions. Upon compression, a LS state of Fe_B_ is also observed [Fig. 3(d)]. In the meanwhile, Fe_3_S_4_ keeps metallic in the high-pressure CS phase throughout the calculated pressure range.

## Discussion

In order to identify the valence state of iron cations in the CS structure, we have calculated the charge density on the FIM-CS (020) section at 3.7 GPa (see Supplementary Figure S3). The calculation illustrates the strong covalence between iron and sulfur due to the existence of high charge density in-between Fe-S bonds. It can be also observed that the distribution of charge around S near to Fe_A_ is more regular than that of S located near Fe_B_ (top-left corner or bottom-right corner), implying that Fe_B_ possesses a stronger polarization than Fe_A_ does. This implies that Fe_B_ possesses a smaller radius or higher chemical valence compared with Fe_A_, either of which indicates that Fe^3+^ occupies the B-site while Fe^2+^ occupies the A-site in the CS structure. Therefore, we suggest a redistribution of iron cations between A-sites and B-sites at the SP-CS transition. In such a case, Fe^2+^ and Fe^3+^ occupy 2*a* (A-site) and 4*i* (B-site) positions, respectively, in the CS structure contrary to the low-pressure inverse spinel structure, where Fe^2+^ and half of Fe^3+^ occupy 16*d* (B-site) positions while the rest of Fe^3+^ occupy 8*a* (A-site) positions. Moreover, considering the magnetic properties of Fe_3_S_4_ displayed in Fig. 2, we propose that the HS-LS transitions of Fe^2+^, occupying A-site, and subsequently Fe^3+^, occupying B-site, in the high-pressure CS phase occur at 8.5 GPa and 17 GPa, respectively. The above-mentioned abrupt changes (local environment of Fe^3+^ at the SP-CS transition and spin state of iron cations) are significant and therefore could be detected by Mössbauer spectroscopy. For example, an increase of coordination number of Fe^3+^ at the SP-CS transition would lead to an abrupt increase of isomer shift. In addition, the spin transitions of iron cations would result in a discontinuous decrease of magnetic hyperfine field and isomer shift^43^,^44^. At the same time, the quadrupole splitting of Fe^2+^ might disappear while that of Fe^3+^ would increase discontinuously at the HS-LS spin transition^44^,^45^. However, the accurate description of changes of hyperfine interaction parameters for Fe_3_S_4_ under high pressure requires further investigation due to the different nature of Fe-S and Fe-O bonds.

**Figure 4:**
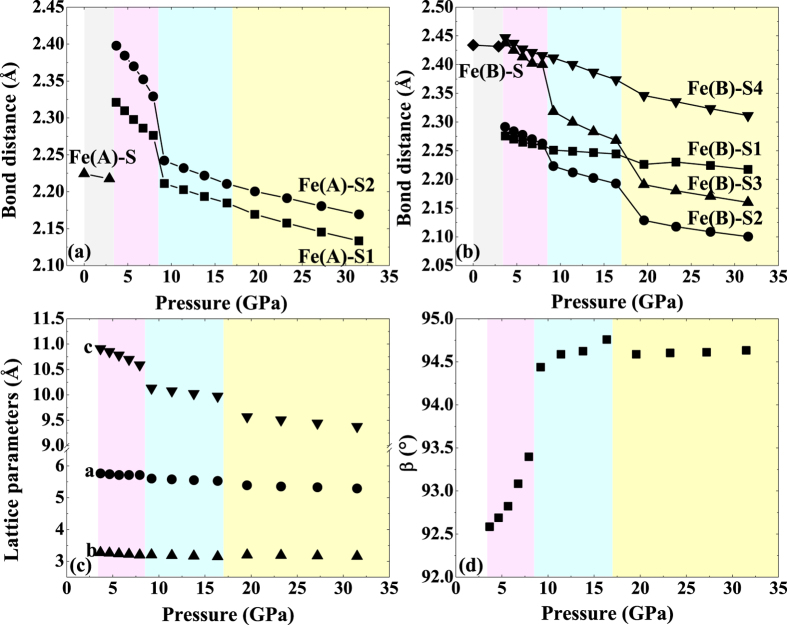
Calculated results of bond distances and lattice parameters of different Fe_3_S_4_ phases as a function of pressure. (**a**) Fe_A_-S bond distances and (**b**) Fe_B_-S bond distances in FIM-SP, FIM-CS, FM1-CS and FM2-CS phases as a function of pressure. (**c**) axis and (**d**) *β* angle of FIM-CS, FM1-CS and FM2-CS phases as a function of pressure.

The compressions of bond distances and lattice parameters of different phases as a function of pressure are shown in Fig. 4. The redistribution of iron cations between A-sites and B-sites leads to abrupt changes of Fe-S bonds at the SP-CS transition at 3.4 GPa [Fig. 4(a) and (b)]. Upon compression, both Fe^2+^-S1 and Fe^2+^-S2 bonds decrease abruptly at 8.5 GPa while all Fe^3+^-S bonds decrease discontinuously at 17 GPa, corresponding to the above-mentioned HS-LS transition sequence of iron cations. It should be noted that although the Fe^3+^-S2 and Fe^3+^-S3 bonds change abruptly at 8.5 GPa, we do not consider these to be due to the spin transition of Fe^3+^. Instead, the HS-LS transition of Fe^2+^ with abrupt decrease in the radius of Fe^2+^ results in a distortion of CS structure, causing some of Fe^3+^-S bonds to change discontinuously. Figure 4(c) presents the fact that the compression of the CS phase is highly anisotropic, i.e., the compressibility of *c*-axis is larger than that of *a*-axis or *b*-axis. The spin transitions of Fe^2+^ and Fe^3+^ cause an abrupt reduction of *c*-axis at 8.5 GPa and 17 GPa, respectively while these transitions affect *a*-axis and *b*-axis only slightly. As shown in Figure S1(b), layers of Fe^2+^ and Fe^3+^ alternate with a cation ordering of Fe^2+^- Fe^3+^- Fe^2+^- Fe^3+^- Fe^2+^ along the *c*-axis in the CS structure. Therefore, the decrease in radius of either Fe^2+^ or Fe^3+^ could exert an influence on *c*-axis. The compression coefficients of the *c*-axis are −6.92 × 10^−3^ GPa^−1^ for FIM-CS phase, −2.18 × 10^−3^ GPa^−1^ for FM1-CS phase and −1.70 × 10^−3^ GPa^−1^ for FM2-CS phase, indicating that the spin transitions of iron make the *c*-axis less compressible. Additionally, the *β* angle increases rapidly from 3.4 GPa to 8.5 GPa, illustrating a higher degree of distortion [Fig. 4 (d)]. This is in agreement with our analysis above.

**Figure 5:**
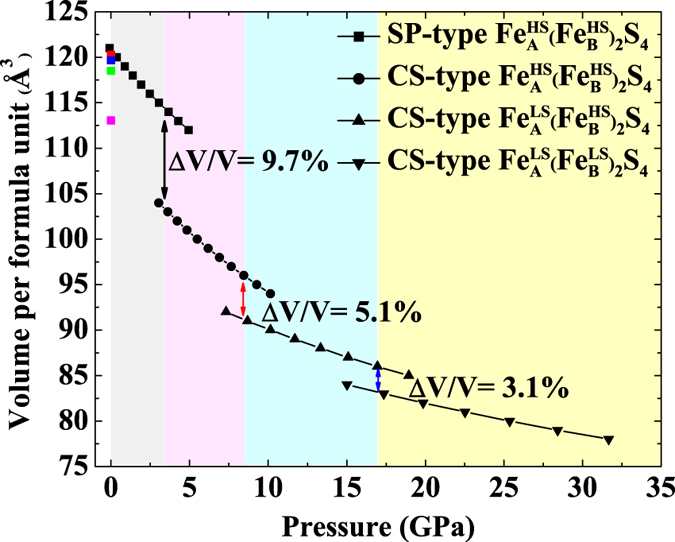
Calculated results of volume per formula unit of different Fe_3_S_4_ phases as a function of pressure. The solid lines segments are fitted by the third-order Birch-Murnaghan Equation of State. The volume collapse of each phase transition is marked. HS and LS represent high-spin and low-spin states, respectively. The previous data marked with red (Exp.), blue (Exp.), green (The.) and pink (The.) points are extracted from refs 38,40 and 42, respectively for comparison.

Figure 5 displays the compressions of volumes of different phases as a function of pressure, and the corresponding parameters have been listed in Table 1. The *K*_*0*_ of FIM-SP phase is in agreement with previous calculation of 62.8 GPa^42^. A smaller *K*_*0*_ of FIM-CS phase, 46.6 GPa, compared to that of FIM-SP phase is obtained in our calculations. This relation between spinel structure and high-pressure post-spinel structure is quite typical and the intrinsic mechanism needs further exploration^10^,^15^. The *K*_*0*_ of FM1-CS and FM2-CS phases increase significantly. Moreover, these three phase transitions, FIM-SP to FIM-CS, FIM-CS to FM1-CS and FM1-CS to FM2-CS, are all first-order with volume reductions of 9.7% at 3.4 GPa, 5.1% at 8.5 GPa and 3.1% at 17 GPa, respectively. An increase of coordination number of Fe^3+^ from four to six mainly contributes to the 9.1% volume collapse. Although the number of Fe^3+^ is twice as that of Fe^2+^ in the chemical formula Fe_3_S_4_, the volume collapse at 8.5 GPa is larger than that at 17 GPa. Both the decrease in radius of Fe^2+^ due to the spin transition and consequently a structural distortion most likely account for the seemingly anomalous volume reduction.

Since the radius of S^2−^ is larger compared to that of O^2−^, the transformation from SP structure to CM-, CT- or CF-type structure for many AB_2_S_4_ compounds is energetically unfavorable. Indeed, it has been reported that many of SP-type transition-metal AB_2_S_4_ compounds transform into CS structure, such as MnCr_2_S_4_, FeCr_2_S_4_ and CoCr_2_S_4_^15^,^46^. The SP-CS phase transition is reconstructive, where the sulfur sub-lattice changes from an fcc to an hcp arrangement for lower energy. It is worthwhile to mention that previous investigations on FeCr_2_S_4_ have shown that the sample is composed solely of the CS phase upon decompression to ambient pressure, indicating an irreversible phase transition^15^. Recent studies on ZnCr_2_Se_4_ under high pressure have confirmed a reversible SP-CS phase transition at 17 GPa by XRD, however^16^. Whether or not the CS phase of Fe_3_S_4_ can be pressure-quenched needs confirmation by experimental methods.

For high-pressure polymorph of Fe_3_O_4_, an occupation of Fe^2+^ in A-sites in either CM phase or CT phase is presumed. However, recent theoretical studies have shown that the total electron numbers of A-sites and B-sites remain unchanged in Fe_3_O_4_ with increasing pressure. The study therefore concluded that the high-pressure phases of Fe_3_O_4_ still possess the chemical formula as Fe_3_O_4_ at ambient conditions^24^. As for Fe_3_S_4_ in our studies, the redistribution of iron cations between A-sites and B-sites occurs at the SP-CS transition. The redistributed occupations of iron cations compared to low- and high-pressure phases of Fe_3_O_4_ discussed above account for different magnetic properties under high pressure in terms of the spin state of iron cations. In addition to the spin transition of Fe^2+^, Ju *et al*.^24^ observed that Fe^3+^ on both A-sites and B-sites underwent a HS-IS transition in the CM-type Fe_3_O_4_ between 50 and 60 GPa in Fe_3_O_4_. However, this claim is not necessarily supported by their calculated sub-lattice magnetic moments or bond distances. In particular, the calculations predicted that the net magnetic moment of Fe_3_O_4_ is lost at 65 GPa and recovered to a nonzero value after the CM-CT transition above 65 GPa, implying Fe_3_O_4_ can be magnetized in the deep lower mantle. This was caused by the fact that Fe^3+^ on the A-site and B-site were not identical. Specifically, Fe^3+^ on the B-site possesses a lower IS-LS transition pressure than Fe^3+^ on the A-site does. In Fe_3_S_4_, both HS and LS state of iron occur under different conditions while the IS state of Fe^2+^ or Fe^3+^ is not observed in our calculations. Although the net magnetic moment changes differently as a function of pressure with respect to the results obtained by Ju *et al*.^24^ due to different occupations of iron cations, the intrinsic spin transition sequence, where Fe^2+^ possesses a lower spin transition pressure and Fe^3+^ possesses a higher spin transition pressure, is basically in agreement with the exception for the absence of an IS state of iron cations in our calculations. The present studies on Fe_3_S_4_, a sulphide counterpart of Fe_3_O_4_, may contribute to provide details towards a more comprehensive view towards Fe_3_O_4_. Additionally, compared to previous studies on Fe_3_O_4_, our calculations predict a FM1-CS phase (CS-type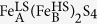) with a significantly positive jump in the net magnetic moment at the transition, which is even higher than that of SP-type Fe_3_S_4_ at ambient pressure. If such a prediction could be confirmed by experiments, it would be also of interest for exploring new magnetic materials.

## Conclusions

In conclusion, the high-pressure behavior of Fe_3_S_4_ has been studied based on first-principle density functional calculations. A first-order phase transition from the SP-type structure to a monoclinic CS-type structure, rather than to an orthorhombic structure, at 3.4 GPa has been established. This reconstructive transition leads to an increase in the coordination number of Fe^3+^ from four to six with a 9.7% volume reduction and a metallic nature of the high-pressure phase of Fe_3_S_4_. According to the calculated charge density of CS-Fe_3_S_4_ as well as bond distances of Fe with respect to S as a function of pressure, a redistribution of iron cations with occupation of Fe^2+^ on A-sites and all of Fe^3+^ on B-sites in the CS-type structure at 3.4 GPa is observed, which has a significant effect on the magnetic properties of high-pressure Fe_3_S_4_ upon further compression. The calculations predict HS-LS transitions of Fe^2+^ and subsequently Fe^3+^, where an abrupt decrease in magnetic moments at 8.5 GPa and 17 GPa result in a volume collapse of 5.1% and 3.1%, respectively. Finally, the Equation of State for different phases of Fe_3_S_4_ are also determined.

## Methods

Five candidate structures (SP, CT, CM, CF and CS) described in the literature were considered for Fe_3_S_4_ in our simulation. First-principle calculations based on density functional theory were performed using the projected augmented wave (PAW) method implemented in Vienna *ab-initio* simulation package (VASP)^47^,^48^,^49^. The Perdew-Burke-Ernzerhof (PBE) version of the generalized gradient approximations (GGA) was selected to treat the exchange correlation potential^50^. The kinetic energy cut-off was set to 600 eV. The energy convergence criterion for the electronic self-consistent calculation was 10^−4^ eV. A DFT + *U* method was introduced in the simulation to correctly describe the strong electronic correlation^51^. *U* = 2.5 eV and *J* = 1 eV were applied into all iron in all GGA + *U* calculations (see Supplementary text and Figure S2). The spin-polarization of iron without spin-orbit coupling was included in calculations to obtain the accurate cell parameters and energies. For SP, CT, CM and CF phases, ferrimagnetic (FIM), ferromagnetic (FM) and anti-ferromagnetic (AFM) structures were included. For CS phase, FIM, FM, non-magnetic (NM), and three AFM (AFM1, AFM2 and AFM3) structures were considered in the calculation. The magnetic arrangement of AFM3 structure was used from ref. 52, thus a 2 × 1 × 2 supercell was constructed. Computations were performed at various volumes for each crystalline phase. The atomic positions, unit-cell parameters and individual magnetic moments were allowed to relax at each given volume to obtain the minimum total energy. The Monkhorst-Pack scheme was used for Brillouin zone sampling. The k-points grids were set as 4 × 4 × 4 for SP phase, 2 × 2 × 8 for CT phase, 8 × 2 × 2 for CM phase, 2 × 8 × 2 for CF phase and 4 × 8 × 2 for CS phase (2 × 8 × 1 for AFM3 CS phase). The DOS were obtained by the static calculation, utilizing the tetrahedral smearing method with Blöchl corrections. The k-points grids were set as 8 × 8 × 8 for SP phase and 12 × 16 × 8 for CS phase in DOS calculation.

Once the minimum total energies of each phase were obtained at different volumes, they were fitted to the third-order Birch-Murnaghan EoS to compute the volume per formula unit (*V*_0_), bulk modulus (*K*_0_), its pressure derivative (*K*_0_’) and energy (*E*_0_) at zero pressure^53^,^54^. In addition, the enthalpy (*H* = *E* + *PV*) of each phase was compared with each other to identify the most stable structure under the given pressure.

## Additional Information

**How to cite this article**: Huang, S. *et al*. Pressure-induced structural and spin transitions of Fe_3_S_4_. *Sci. Rep.* **7**, 46334; doi: 10.1038/srep46334 (2017).

**Publisher's note:** Springer Nature remains neutral with regard to jurisdictional claims in published maps and institutional affiliations.

## Supplementary Material

Supplementary Information

## Figures and Tables

**Table 1:** The calculated parameters of the third-order Birch-Murnaghan Equation of State (energy per formula unit *E*_*0*_, volume per formula unit *V*_0_, bulk modulus *K*_0_, its pressure derivative *K*_0_’ at zero pressure) of different Fe_3_S_4_ phases.

Phase	*E*_*0*_/f.u. (eV)	*V*_*0*_/f.u. (Å^3^)	*K*_*0*_ (GPa)	*K*_*0*_’
FIM-SP	−40.71	120.8	56.6	3.8
FM-SP	−39.92	116.5	48.0	5.4
AFM-SP	−40.30	120.4	43.2	5.3
FIM -CT	−39.62	113.9	55.1	3.5
FM -CT	−39.21	112.5	41.7	5.2
AFM-CT	−39.74	117.7	44.6	3.7
FIM -CM	−39.62	113.7	56.3	3.4
FM -CM	−39.22	112.6	41.8	5.1
AFM-CM	−39.75	117.8	45.0	3.7
FIM -CF	−39.47	113.1	76.3	3.6
FM -CF	−39.39	114.8	37.5	3.3
AFM-CF	−39.65	113.5	57.0	3.5
FIM -CS	−40.48	110.2	46.6	3.9
FM1-CS	−40.07	98.7	86.9	5.0
FM2-CS	−39.64	92.7	118.6	5.0
AFM1-CS	−40.34	116.1	53.7	4.0
AFM2-CS	−40.22	114.5	30.0	7.2
AFM3-CS	−40.28	112.9	43.3	4 (fixed)
NM -CS	−39.58	92.8	117.1	4.9

## References

[b1] Ringwood A. E. Composition and Petrology of The Earth’s Mantle. 618 (McGraw-Hill, 1975).

[b2] Katsura T. & Ito E. The system Mg_2_SiO_4_-Fe_2_SiO_4_ at high pressures and temperatures: Precise determination of stabilities of olivine, modified spinel, and spinel. J. Geophys. Res. [Solid Earth] 94, 15663–15670 (1989).

[b3] Hemberger J. . Relaxor ferroelectricity and colossal magnetocapacitive coupling in ferromagnetic CdCr_2_S_4_. Nature 434, 364–367 (2005). 15772656 10.1038/nature03348

[b4] Weber S. . Colossal Magnetocapacitance and colossal magnetoresistance in HgCr_2_S_4_. Phys. Rev. Lett. 96, 157202 (2006). 16712192 10.1103/PhysRevLett.96.157202

[b5] Yokaichiya F. . Spin-driven phase transitions in ZnCr_2_Se_4_ and ZnCr_2_S_4_ probed by high-resolution synchrotron x-ray and neutron powder diffraction. Phys. Rev. B 79, 064423 (2009).

[b6] Bordács S. . Magnetic-order-induced crystal symmetry lowering in ACr_2_O_4_ ferrimagnetic spinels. Phys. Rev. Lett. 103, 077205 (2009). 19792683 10.1103/PhysRevLett.103.077205

[b7] Ono S., Kikegawa T. & Ohishi Y. The stability and compressibility of MgAl_2_O_4_ high-pressure polymorphs. Phys. Chem. Mineral. 33, 200–206 (2006).

[b8] Fei Y., Frost D. J., Mao H. K., Prewitt C. T. & Haeusermann D. *In situ* structure determination of the high-pressure phase of Fe_3_O_4_. Am. Mineral. 84, 203–206 (1999).

[b9] Haavik C., Stølen S., Fjellvåg H., Hanfland M. & Häusermann D. Equation of state of magnetite and its high-pressure modification: Thermodynamics of the Fe-O system at high pressure. Am. Mineral. 85, 514–523 (2000).

[b10] Ye L. . Compressibilities of MnFe_2_O_4_ polymorphs. Phys. Chem. Mineral. 42, 569–577 (2015).

[b11] Ferrari S. . *In-situ* high-pressure x-ray diffraction study of zinc ferrite nanoparticles. Solid State Sci. 56, 68–72 (2016).

[b12] Wang Z. . High-pressure x-ray diffraction and Raman spectroscopic studies of the tetragonal spinel CoFe_2_O_4_. Phys. Rev. B 68, 094101 (2003).

[b13] Kyono A. . High-pressure behavior of cuprospinel CuFe_2_O_4_: Influence of the Jahn-Teller effect on the spinel structure. Am. Mineral. 100, 1752–1761 (2015).

[b14] Efthimiopoulos I. . Pressure-induced transition in the multiferroic CoCr_2_O_4_ spinel. Phys. Rev. B 92, 064108 (2015).

[b15] Amiel Y. . Intricate relationship between pressure-induced electronic and structural transformations in FeCr_2_S_4_. Phys. Rev. B 84, 224114 (2011).

[b16] Efthimiopoulos I. . Structural transition in the magnetoelectric ZnCr_2_Se_4_ spinel under pressure. Phys. Rev. B 93, 174103 (2016).

[b17] Mao H. K., Takahashi T., Bassett W. A., Kinsland G. L. & Merrill L. Isothermal compression of magnetite to 320 KB. J. Geophys. Res. [Solid Earth] 79, 1165–1170 (1974).

[b18] Dubrovinsky L. S. . The structure of the metallic high-pressure Fe_3_O_4_ polymorph: experimental and theoretical study. J. Phys.: Condens. Matter 15, 7697–7706 (2003).

[b19] Lazor P., Shebanova O. N. & Annersten H. High-pressure study of stability of magnetite by thermodynamic analysis and synchrotron X-ray diffraction. J. Geophys. Res. [Solid Earth] 109, B05201 (2004).

[b20] Rozenberg G. K. . Structural characterization of temperature- and pressure-induced inverse↔normal spinel transformation in magnetite. Phys. Rev. B 75, 020102(R) (2007).

[b21] Glazyrin K. . Effect of high pressure on the crystal structure and electronic properties of magnetite below 25 GPa. Am. Mineral. 97, 128–133 (2012).

[b22] Ding Y. . Novel pressure-induced magnetic transition in magnetite (Fe_3_O_4_). Phys. Rev. Lett. 100, 045508 (2008). 18352301 10.1103/PhysRevLett.100.045508

[b23] Baudelet F. . Absence of abrupt pressure-induced magnetic transitions in magnetite. Phys. Rev. B 82, 140412(R) (2010).

[b24] Ju S., Cai T. Y., Lu H. S. & Gong C. D. Pressure-induced crystal structure and spin-state transitions in magnetite (Fe_3_O_4_). J. Am. Chem. Soc. 134, 13780–13786 (2012). 22823905 10.1021/ja305167h

[b25] Bengtson A., Morgan D. & Becker U. Spin state of iron in Fe_3_O_4_ magnetite and h-Fe_3_O_4_. Phys. Rev. B 87, 155141 (2013).

[b26] Cao F. . 3D Fe_3_S_4_ flower-like microspheres: high-yield synthesis via a biomolecule-assisted solution approach, their electrical, magnetic and electrochemical hydrogen storage properties. Dalton Trans. 42, 9246–9252 (2009). 10.1039/b912569h 20449202

[b27] Zhang W., Cheng Y., Han D. & Han S. The hydrogen storage properties of MgH_2_–Fe_3_S_4_ composites. Energy 93, 625–630 (2015).

[b28] Paolella A. . Charge transport and electrochemical properties of colloidal greigite (Fe_3_S_4_) nanoplatelets. Chem. Mater. 23, 3762–3768 (2011).

[b29] Roldan A. & de Leeuw N. H. Catalytic water dissociation by greigite Fe_3_S_4_ surfaces: density functional theory study. Proc. R. Soc. A 472, 20160080 (2016). 27274698 10.1098/rspa.2016.0080 PMC4892285

[b30] Santos-Carballal D., Roldan A. & de Leeuw N. H. Early oxidation processes on the greigite Fe_3_S_4_ (001) surface by water: A density functional theory study. J. Phys. Chem. C, 120, 8616–8629 (2016).

[b31] Roberts A. P. & Weaver R. Multiple mechanisms of remagnetization involving sedimentary greigite (Fe_3_S_4_). Earth Planet. Sci. Lett. 231, 263–277 (2005).

[b32] Roberts A. P., Chang L., Rowan C. J., Horng C. S. & Florindo F. Magnetic properties of sedimentary greigite (Fe_3_S_4_): An update. Rev. Geophys. 49, RG1002 (2011).

[b33] Coey J. M. D., Spender M. R. & Morrish A. H. The magnetic structure of the spinel Fe_3_S_4_. Solid State Commun. 8, 1605–1608 (1970).

[b34] Spender M. R., Coey J. M. D. & Morrish A. H. The magnetic properties and Mössbauer spectra of synthetic samples of Fe_3_S_4_. Can. J. Phys. 50, 2313–2326 (1972).

[b35] Hoffmann V. Greigite (Fe_3_S_4_): magnetic properties and first domain observations. Phys. Earth Planet. Inter. 70, 288–301 (1992).

[b36] Chang L. . Magnetic characteristics of synthetic pseudo-single-domain and multi-domain greigite (Fe_3_S_4_). Geophys. Res. Lett. 34, L24304, (2007).

[b37] Chang L. . Fundamental magnetic parameters from pure synthetic greigite (Fe_3_S_4_). J. Geophys. Res.: [Solid Earth] 113, B06104 (2008).

[b38] Chang L. . Magnetic structure of greigite (Fe_3_S_4_) probed by neutron powder diffraction and polarized neutron diffraction. J. Geophys. Res.: [Solid Earth] 114, B07101 (2009).

[b39] Verwey E. J. W. Electronic conduction of magnetite (Fe_3_O_4_) and its transition point at low temperatures. Nature 144, 327–328 (1939).

[b40] Devey A. J., Grau-Crespo R. & de Leeuw N. H. Electronic and magnetic structure of Fe_3_S_4_: GGA+U investigation. Phys. Rev. B 79, 195126 (2009).

[b41] Wu M., John S. T. & Pan Y. Electronic structures of greigite (Fe_3_S_4_): A hybrid functional study and prediction for a Verwey transition. Sci. Rep. 6, 21637 (2016). 26869147 10.1038/srep21637 PMC4751502

[b42] Roldan A., Santos-Carballal D. & de Leeuw N. H. A comparative DFT study of the mechanical and electronic properties of greigite Fe_3_S_4_ and magnetite Fe_3_O_4_. J. Chem. Phys. 138, 204712 (2013). 23742505 10.1063/1.4807614

[b43] Lin J. F. . Magnetic transition and sound velocities of Fe_3_S at high pressure: Implications for Earth and planetary cores. Earth Planet. Sci. Lett. 226, 33–40 (2004).

[b44] Xu W. M., Machavariani G. Y., Rozenberg G. K. & Pasternak M. P. Mössbauer and resistivity studies of the magnetic and electronic properties of the high-pressure phase of Fe_3_O_4_. Phys. Rev. B 70, 174106 (2004).

[b45] Lin J. F., Speziale S., Mao Z. & Marquardt H. Effects of the electronic spin transitions of iron in lower mantle minerals: implications for deep mantle geophysics and geochemistry. Rev. Geophys. 51, 244–275 (2013).

[b46] Tressler R. E., Hummel F. A. & Stubican V. S. Pressure-temperature study of sulfospinels. J. Am. Ceram. Soc. 51, 648–651 (1968).

[b47] Blöchl P. E. Projector augmented-wave method. *Phys*. Rev. B 50, 17953–17979 (1994). 10.1103/physrevb.50.17953 9976227

[b48] Kresse G. & Joubert D. From ultrasoft pseudopotentials to the projector augmented-wave method. Phys. Rev. B 59, 1758–1775 (1999).

[b49] Kresse G. & Furthmüller J. Efficient iterative schemes for *ab initio* total-energy calculations using a plane-wave basis set. Phys. Rev. B 54, 11169–11186 (1996). 10.1103/physrevb.54.11169 9984901

[b50] Perdew J. P., Burke K. & Ernzerhof M. Generalized gradient approximation made simple. Phys. Rev. Lett. 77, 3865–3868 (1996). 10062328 10.1103/PhysRevLett.77.3865

[b51] Dudarev S. L., Botton G. A., Savrasov S. Y., Humphreys C. J. & Sutton A. P. Electron-energy-loss spectra and the structural stability of nickel oxide: An LSDA+ U study. Phys. Rev. B 57, 1505–1509 (1998).

[b52] Min B. I., Baik S. S., Choi H. C., Kwon S. K. & Kang J. S. Electronic structures of magnetic semiconductors FeCr_2_Se_4_ and Fe_0.5_Cu_0.5_Cr_2_Se_4_. New J. Phys. 10, 055014 (2008).

[b53] Birch F. Finite Elastic strain of cubic crystals. Phys. Rev. 71, 809–824 (1947).

[b54] Murnaghan F. D. The compressibility of media under extreme pressures. Proc. Natl. Acad. Sci. USA 30, 244–247 (1944). 16588651 10.1073/pnas.30.9.244 PMC1078704

