# Supplementary material for: Pressure-induced structural and spin transitions of Fe3S4
Source: Sci Rep. 2017 Apr 12;7:46334. doi: 10.1038/srep46334 (PMC5389354; doi:10.1038/srep46334)
Supplement: Supplementary Information [file srep46334-s1.pdf]

Supplementary Material

Pressure-induced structural and spin transitions of Fe_3S_4

Shengxuan Huang,[1] Duan Kang,[1] Xiang Wu,[2,*] Jingjing Niu,[1] and Shan Qin[1]

[1]Key Laboratory of Orogenic Belts and Crustal Evolution, MOE, Peking University and School of Earth and Space Sciences, Peking University, Beijing 100871, P. R. China

[2]State key laboratory of geological processes and mineral resources, China University of Geosciences (Wuhan), 430074, P. R. China

[*] Corresponding author: wuxiang@cug.edu.cn

Supplementary Text

The choice of the Hubbard *U* parameter

The calculated lattice parameter and sub-lattice magnetic moments of SP-type Fe_3S_4 at ambient pressure by the GGA method were 9.491 Å, -1.83 μ_B for Fe_A and 1.96 μ_B for Fe_B, respectively, which were much lower than the experimental results (-3.16 μ_B for Fe_A and 3.24 μ_B for Fe_B) obtained by NPD[1]. A DFT + *U* method, therefore, was introduced in the simulation to correctly describe the strong electronic correlation[2].

The correction to the GGA energy considering the Hubbard *U* parameter is generally expressed as the following functional[2]

$$E_{\mathrm{GGA}+U} = E_{\mathrm{GGA}} + \frac{U-J}{2}\sum_{\sigma}\mathrm{Tr}[\rho^{\sigma} - \rho^{\sigma}\rho^{\sigma}],$$

where *J* is an approximation of Hund's exchange parameter and ρ^{σ} is the on-site density matrix. Such a functional indicates that the parameter $\frac{U-J}{2}$ (usually expressed as U_{eff}) determines the correction to the GGA energy. In addition, the exchange parameter *J* is almost constant at ~ 1 eV[3]. It is worthwhile to mention that the *U* = 1 eV case then represents the GGA limit given *J* = 1 eV.

Thus, in order to determine a reasonable *U* value compatible with our present system in the simulation, we varied the Hubbard *U* parameter in the range from 1 to 5 eV at a fixed value of 1 eV for *J* (Fig. S2). The calculated lattice parameter and absolute value of sub-lattice magnetic moments of SP-type Fe_3S_4 at ambient pressure increased with increasing *U* value except the case with *U* = 5 eV, in which the magnetic moment of Fe_B dropped significantly. We have chosen experimental NPD results at 10 K (black and red horizontal dashed lines) as our criterion for determining *U* because the temperature can strongly affect magnetic moments of SP-type Fe_3S_4[1]. Finally, *U* = 2.5 eV and *J* = 1 eV were determined and applied to all iron atoms in the GGA + *U* calculations. We then calculated electronic DOS of each equivalent atom site of SP-type Fe_3S_4 at ambient pressure [Fig. 3(a)]. A half-metallic character for SP-type Fe_3S_4 at ambient pressure was observed in agreement with previous simulations by the GGA + *U* method[4]. Therefore, the choice of Hubbard *U* parameter in the present simulation was reasonable and self-consistent.

Previous studies have systematically investigated electronic correlation effects in transition-metal sulfides[5]. They have found that the introduction of DFT + *U* method yields improved predictions for properties of transition-metal sulfides. The magnetic moment of FeS, for example,

could be better predicted even by the introduction of a smaller value of U compared with other transition-metal sulfides. In particular, Devey *et al.*[4] have recently discussed the influence of U on properties of SP-type Fe_3S_4 and concluded that an accurate description of all properties of SP-type Fe_3S_4 requires a U_{eff} value of ~1 eV. That means a U value close to our choice of 3 eV if J = 1 eV. Moreover, the PW91 version of GGA was selected as exchange-correlation functional in their calculations while the PBE version of GGA was used in our studies. Thus, a slight difference between two chosen U values is very common.

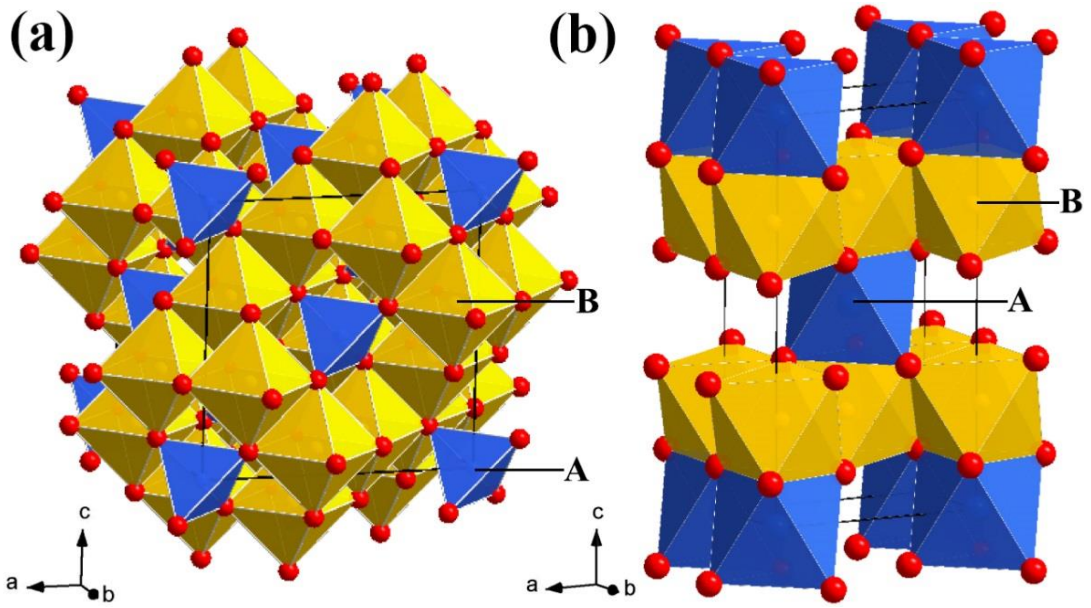

Fig. S1. The spinel-type ($Fd\bar{3}m$, Z = 8, SP) (a) and Cr_3S_4-type ($I2/m$, Z = 2, CS) (b) structures of AB_2X_4 compounds. (a) The tetrahedral A-sites and octahedral B-sites in SP-type structure are shown in blue and yellow objects, respectively. (b) The octahedral A-sites and octahedral B-sites in CS-type structure are shown in blue and yellow objects, respectively.

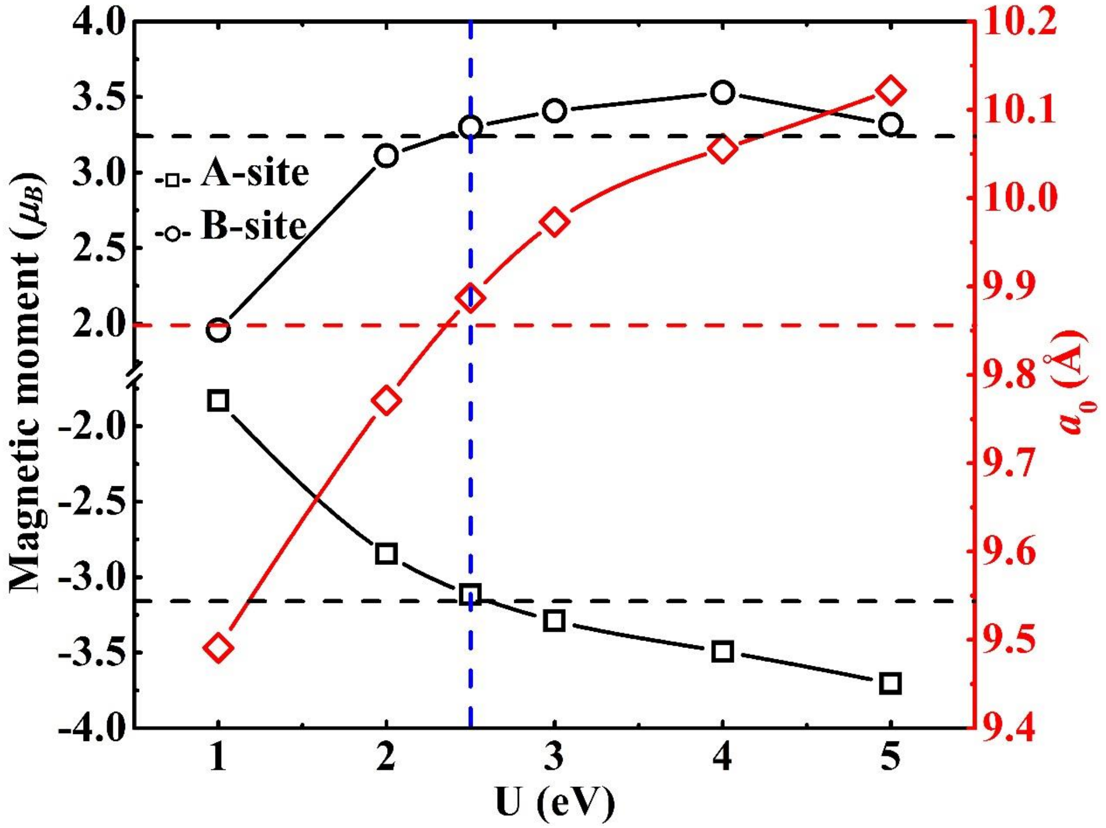

Fig. S2. The calculated sub-lattice magnetic moments of A-site (black open squares) and B-site (black open circles) and lattice parameter (red open diamonds) of SP-type Fe_3S_4 at ambient pressure as a function pf Hubbard *U* parameter. The black and red horizontal dashed lines are experimental data extracted from Ref. [1]. The blue vertical dashed line represents the *U* value used in our studies.

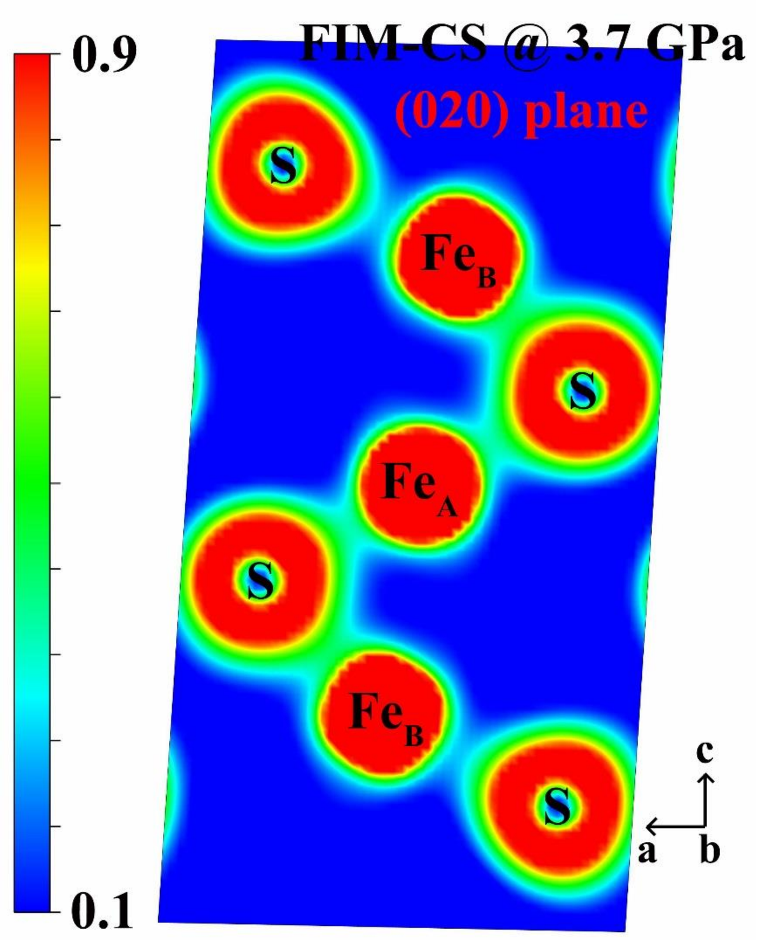

Fig. S3. The calculated charge density on the FIM-CS (020) section at 3.7 GPa by GGA + *U* method. The occupations of Fe_A, Fe_B and S are marked by black numbers.

Supplementary References:

1. Chang, L. *et al*. Magnetic structure of greigite (Fe_3S_4) probed by neutron powder diffraction and polarized neutron diffraction. *J. Geophys. Res.: [Solid Earth]* **114,** B07101 (2009).
2. Dudarev, S. L., Botton, G. A., Savrasov, S. Y., Humphreys, C. J. & Sutton, A. P. Electron-energy-loss spectra and the structural stability of nickel oxide: An LSDA+U study. *Phys. Rev. B* **57,** 1505-1509 (1998).
3. Solovyev, I. V., Dederichs, P. H., & Anisimov, V. I. Corrected atomic limit in the local-density approximation and the electronic structure of d impurities in Rb. *Phys. Rev. B* **50**, 16861-16871 (1994).
4. Devey, A. J., Grau-Crespo, R. & de Leeuw, N. H. Electronic and magnetic structure of Fe_3S_4: GGA+U investigation. *Phys. Rev. B* **79,** 195126 (2009).
5. Rohrbach, A., Hafner, J., & Kresse, G. Electronic correlation effects in transition-metal sulfides. *J. Phys.: Condens. Matter* **15**, 979-996 (2003).
